# Beam orientation optimization for coherent X-ray scattering from distributed deep targets

**DOI:** 10.1186/s12938-021-00928-x

**Published:** 2021-09-15

**Authors:** Sophya Breedlove, Aldo Badano

**Affiliations:** Division of Imaging, Diagnostics, and Software Reliability, Office of Science and Engineering Laboratories, Center for Devices and Radiological Health, Food and Drug Administration, 10903 New Hampshire Avenue, Silver Spring, MD 20993 USA

## Abstract

**Background:**

Amyloid deposits in the temporal and frontal lobes in patients with Alzheimer’s disease make them potential targets to aid in early diagnosis. Recently, spectral small-angle X-ray scattering techniques have been proposed for interrogating deep targets such as amyloid plaques.

**Results:**

We describe an optimization approach for the orientation of beams for deep target characterization. The model predicts the main features of scattering profiles from targets with varying shape, size and location. We found that increasing target size introduced additional smearing due to location uncertainty, and incidence angle affected the scattering profile by altering the path length or effective target size. For temporal and frontal lobe targets, beam effectiveness varied up to 2 orders of magnitude.

**Conclusions:**

Beam orientation optimization might allow for patient-specific optimal paths for improved signal characterization.

## Background

Beam orientation optimization has been used in X-ray procedures, including intensity-modulated radiation therapy for cancer patients and C-arm imaging. Beam angle optimization and intensity optimization techniques are implemented in intensity-modulated radiation therapy to ensure sufficient radiation doses are delivered to the target volume, while minimizing unnecessary radiation exposure to critical radiosensitive structures. Pose optimization is used for C-arm imaging devices to reduce radiation exposure to patients and staff while maintaining sufficient quality in the acquired images [1]. Beam orientation for small-angle, X-ray scattering (SAXS) in the human head would be advantageous because of its potential applications in detection of amyloid plaques in the brain as an aid in the early diagnosis of neurodegenerative diseases such as Alzheimer’s disease (AD). Traditionally, SAXS is used for small targets, but recent studies have proposed using SAXS to interrogate deep targets in large objects [2]. We assess the feasibility of optimizing beam orientation to obtain useful SAXS signals from regions of preclinical amyloid deposition in the human brain.

Many studies agree that high amyloid deposition occurs in the frontal, temporal, and parietal lobes. The areas of the frontal cortex mainly involved in preclinical deposition are the orbitofrontal [3–5] and inferior frontal regions, as well as the middle frontal and superior frontal regions [4]. Deposition in the temporal cortex mainly occurs in the inferior temporal gyri [4–7], middle temporal gyri [4, 6], and perirhinal cortex (in medial temporal lobe) [8, 9]. Deposition in the parietal cortex occurs in the precuneus (part of the superior parietal region) [3, 5, 10] as well as the parietal operculum (part of the inferior parietal region) [7]. Evidence is also found of preclinical amyloid deposition in the anterior [7] and posterior [3, 5] cingulate gyrus [11]. Some studies disagree about the areas of the earliest amyloid deposition, and one in particular appears to suggest that there is earlier subcortical amyloid deposition rather than cortical, which contradicts previous literature. The study proposed high deposition in some cortical regions (cingulate), but also high amyloid deposition in subcortical regions (pallidum, putamen, and thalamus). One limitation and potential reason for discrepancy in this study was the segmentation software’s decreased accuracy in delineating regions of interest in subcortical regions. In addition, while the study proposed a cutoff value of SUVR for amyloid positivity in cortical regions, it did not define a cutoff value for subcortical regions. [11]

Additional reasons for discrepancies concerning the regions of earliest amyloid deposition include different analytical techniques in each of the studies. Studies differed in their definition of amyloid positivity, with some defining it based on the proportion of regions that exhibited a suprathreshold SUVR signal, while others did not propose cutoff values for SUVR. Some studies estimated AD stage based on the proportion of participants exhibiting amyloid pathology in a given region, implying that the amount of amyloid deposition corresponds to the amount of accumulation time, while other studies based AD stage mainly on the amount of biomarkers for a given participant. Overall, it appears that some of the earliest amyloid deposition in preclinical AD occurs in the basal portions of the frontal and temporal lobes.

In this work, we describe a model of SAXS signals for the optimization of beam orientation in detecting amyloid plaques found in regions of early amyloid deposition, such as the frontal and temporal lobes. To investigate the use of SAXS in large objects such as the human head, we developed a model to predict the X-ray scattering profiles of spherical, ellipsoidal, and arbitrarily shaped 2D targets of varying size for different locations within the object and X-ray incidence angle. To determine the effectiveness of each orientation and allow for patient-specific beam optimization, we considered radiation dose, peak relative scattering intensity, scattering vector smearing, and total relative scattering intensity for each scattering profile.

## Results

### Spherical target

We modeled the scattering signal of a spherical target embedded in a spherical object, representing an amyloid target region embedded in a human brain. This model assumes the attenuation coefficient of the brain is the same as that of the amyloid target, and that the density of amyloid in the rest of the brain is negligible.

**Fig. 1:**
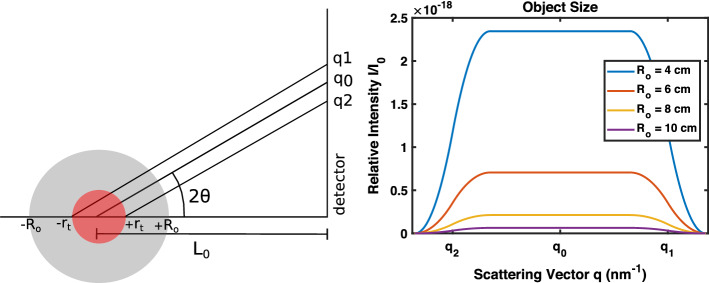
Scattering pattern for a target region embedded within a human head of varying size. Object size $R_o$ varies from 4 to 10 cm, target size $r_t$ is 2 cm, and mean target-to-detector distance $L_0$ is 20 cm

Figure 1 shows scattering signals with diminished intensity due to attenuation effects from the size of the brain (object). While it is clear that the scattering signal has been reduced, this model shows that a signal is still detectable when the target is embedded within a larger object. It also demonstrates that a smaller object size yields greater relative intensity.

**Fig. 2:**
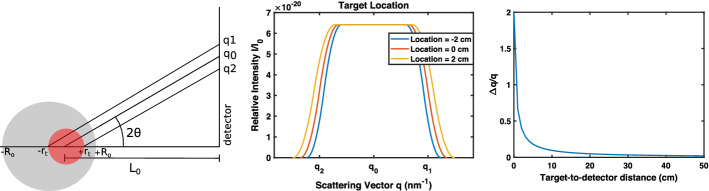
Scattering patterns for a deep target based on target location within an object. The right graph shows maximum relative deviation in *q* in terms of target-to-detector distance to explain the change in smearing based on target location. Object size $R_o$ is 10 cm, target size $r_t$ is 2 cm, and mean target-to-detector distance $L_0$ varies from 18 to 22 cm

When the target is placed at different locations along the path of the X-ray without changing the X-ray incidence angle, there is a small change in intensity due to differences in path length, as shown in Fig. 2, but since scattering angles are small, this difference is also small. The uncertainty in the scattering vector is affected by target location: when the target is closer to the detector, there is more uncertainty in the scattering vector.

The ability to interrogate a deep target from different X-ray incidence angles can sometimes allow for a reduction in attenuation from object size provided the target is not located at the center of the object. The path length through a sphere can be calculated using the chord length formula for a circle, $d = 2 R_o sin \left( C/2 \right)$, where *d* is the path length, $R_o$ is the radius of the object, and *C* is the angle subtended at the center of the sphere by the entry and exit points of the X-ray beam.

**Fig. 3:**
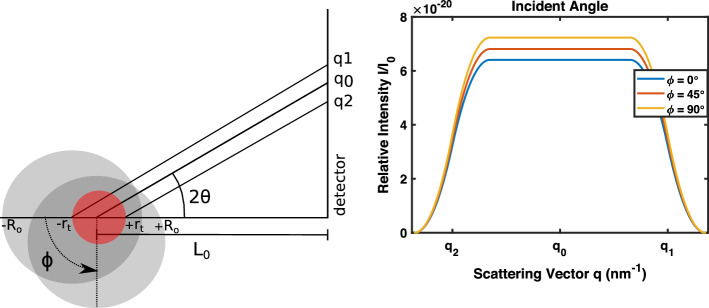
Scattering patterns for a deep target based on the incident angle of the incoming X-ray beam. Object size $R_o$ is 10 cm, target size $r_t$ is 2 cm, and mean target-to-detector distance $L_0$ is 20 cm

This model shows that when the X-ray incidence angle is altered, the scattering signal changes. Figure 3 shows that when the incidence angle results in a smaller path length, the relative intensity of the scattering signal increases.

### Non-spherical target

**Fig. 4:**
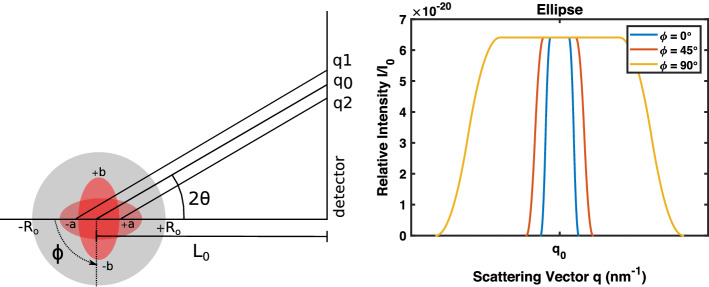
Scattering patterns of an elliptical deep target (minor axis *a* = 1 cm and major axis *b* = 3 cm) has been modeled for different angles of incidence. Object size $R_o$ is 10 cm and mean target-to-detector distance $L_0$ is 20 cm

The frontal and temporal lobe regions can be approximated using ellipsoids. The polar form of an ellipse equation, $r_e^2 = (a b)^2 / [b^2 cos(\phi )^2 + a^2 sin(\phi )^2]$, shows how the radius of the ellipse changes depending on the angle of incidence $\phi$ and the ellipse axes *a* and *b*. Figure 4 demonstrates the change in the uncertainty of the scattering vector based on target shape and X-ray incidence angle. The relative intensity is not affected, since the attenuation through the centered, spherical object is the same at all incidence angles. The uncertainty of the scattering vector is affected based on incidence angle, since the target radius changes, with a larger target radius resulting in more uncertainty in the scattering vector.

### Segmented 2D target

**Fig. 5:**
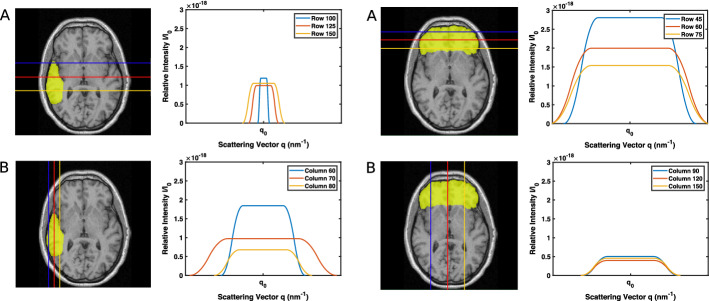
Scattering signals for X-ray paths through a segmented region from a 2D scout image of the temporal (left) and frontal (right) lobe, showing a selection of **A** horizontal and **B** vertical paths through target

We modeled the scattering profile of a segmented 2D brain region for Cartesian grid angle X-ray incidences. 2D image segmentation was performed using the MATLAB Image Segmenter App. Regions of interest were segmented manually to create a binary mask with pixels assigned to be either “target” pixels or “object” pixels using visual guidance from a human brain atlas [12]. Temporal and frontal lobe regions were segmented using this method, with lobe pixels assigned the “target” value and pixels of the surrounding brain and skull regions assigned the “object” value. This manual segmentation technique was sufficient for providing a first proof-of-concept demonstration of the model for an arbitrarily shaped deep target. A selection of the scattering signals for the temporal and frontal lobes is shown in Fig. 5. A measurement for the path length through the object was obtained by counting the number of assigned object pixels within the beam path and converting that to a distance. Similarly, the measurement for the target radius was related to the number of assigned target pixels. Calculations assumed that the location of the target is known, and that target-to-detector distance does not change. The measure of X-ray beam angle effectiveness, $\Omega$, through the temporal target region varied by up to an order of magnitude, and beam effectiveness through the frontal region varied by up to 2 orders of magnitude, as shown in Table 1.

**Table 1:** Peak relative intensity, $\Delta$ q, and $I_t$ values for a selection of paths through a segmented region, corresponding to paths in Fig. 5

Path	$I_p$	$I_t$ ($\text{nm}^{-1}$)	$\Delta$ q ($\text{nm}^{-1}$)	$\Omega$	$\frac{\Omega }{\Omega _{max}}$
Row 100	1.2 e−18	5.3 e−19	0.3	2.7 e−37	0.36
Row 125	1.0 e−18	1.1 e−18	0.8	1.8 e−37	0.23
Row 150	1.1 e−18	1.7 e−18	1.1	2.1 e−37	0.27
Column 60	1.8 e−18	5.9 e−18	2.2	7.7 e−37	1
Column 70	9.7 e−19	5.4 e−18	3.8	1.7 e−37	0.22
Column 80	6.8 e−19	2.5 e−18	2.5	7.6 e−38	0.10
Row 45	2.8 e−18	1.4 e−17	3.4	2.1 e−36	1
Row 60	2.0 e−18	1.2 e−17	4.1	9.4 e−37	0.44
Row 75	1.5 e−18	9.4 e−18	4.1	5.0 e−37	0.24
Column 90	5.0 e−19	1.8 e−18	2.5	3.8 e−38	0.02
Column 120	4.0 e−19	1.5 e−18	2.6	2.2 e−38	0.01
Column 150	4.5 e−19	1.7 e−18	2.6	3.1 e−38	0.01

In a human brain, the variation in brain tissue would provide additional scattering and different linear attenuation coefficients. The additional scattering from brain tissues outside the target region should not affect the results of this model, since the background signal from the tissue outside the target is subtracted from the original scattering signal and therefore only represents the scattering signal from the target region. Additionally, scattering from the brain tissue would occur in a range outside that of amyloid scattering (see Ref. [13]). If the differences in linear attenuation are small, there should be no effect on optimization results, but if the differences are more drastic, optimization results may be affected. The differences in radiosensitivity between bone and neuronal tissues are relatively small, so the assumption that radiation dose is directly related to path length should not affect optimization results, as long as areas with high radiosensitivity are not included as options for optimal beam orientation.

## Discussion

The method described in this work demonstrates the importance of beam orientation optimization for small-angle X-ray scattering and provides an initial approximation to the optimal solution for reducing radiation exposure and increasing SAXS signal conspicuity. It considers absorbed radiation dose in order to reduce unnecessary radiation exposure, which can have harmful side effects. Since high total intensity, high peak relative intensity, and less smearing of the scattering vector result in more easily distinguishable peaks, this beam orientation optimization method should result in a greater likelihood of accurately deciphering SAXS signals to detect amyloid plaques, leading to more accurate diagnoses.

This model accounts for radiosensitive tissues by avoiding sensitive areas and assuming all other regions of the brain have similarly low radiosensitivities. It does not automatically discard paths that intersect sensitive regions. Automation of this process could be achieved by assigning radiosensitivity values for each pixel (or voxel) based on segmented regions and incorporating these values into the figure of merit to determine effective radiation dose.

The assumption of one linear attenuation coefficient for the entire head could skew optimization results, as linear attenuation coefficients vary among regions of the human head and linear attenuation coefficients influence relative scattering intensity. If the differences in linear attenuation coefficients and path lengths through each region are drastic enough, beam orientation optimization results could be affected. A more accurate model of attenuation effects can be developed by segmenting regions with drastically different linear attenuation coefficients, such as the skull, determining the X-ray path length through each segmented region, and incorporating these values into relative intensity calculations.

In this model, amyloid plaques were assumed to be evenly distributed throughout target regions. In reality, there would likely be variable amyloid plaque distribution within a target region. Since relative scattering intensity is modeled as a function of the amyloid density at each point along the target, a non-uniform density can be modeled by segmenting subregions of similar amyloid density and assigning density values to each pixel (or voxel) to calculate the relative scattering intensity at each pixel (or voxel) along the X-ray path. High-density regions within the target would result in a scattering profile with higher intensity values, while low-density regions would result in lower intensity values. Regions of the target that are closer to the detector will affect the scattering profile by altering the intensity corresponding to smaller scattering vector values, while target regions that are further from the detector will alter the intensity values corresponding to larger scattering vector values. The uncertainty in the scattering vector is not affected by density. Once the scattering profile has been obtained by running the model for each subregion, the process and figure of merit for determining the optimal path for a target of uniform density can be applied to the non-uniform target case.

Beam orientation optimization can be performed at all angles for well-defined shapes such as spheres and ellipsoids, but segmentation of arbitrary shapes make assigning pixels to non-Cartesian grid angles more complicated. A method for assigning pixels along a path based on angle and location should be established.

Within a clinical application of beam orientation optimization, targets would be examined using 3D imaging rather than only 2D scout images, necessitating that this beam orientation optimization technique be translated to segmented 3D brain volumes of interest. This requires the use of a segmented 3D scout image and involves assigning values to voxels rather than pixels. While 2D images in this work were segmented using a MATLAB Image Segmenter App, 3D regions of interest can similarly be segmented using the MATLAB Volume Segmenter App and assigning voxel values. Other image processing software including ImageJ in combination with 3D segmentation algorithms could also be used. Due to the time-consuming nature of manually segmenting 3D regions of interest, more advanced 3D segmentation techniques should be used in a clinical implementation. A variety of methodologies and models have been developed for automatic segmentation of specific brain regions (see, for instance, Ref. [14]). Modeling the scattering signal of temporal and frontal target regions in a 2D scout image resulted in beam effectiveness that varied significantly, up to 2 orders of magnitude. This substantial variation in beam effectiveness highlights the importance of beam orientation optimization.

One limitation of the proposed model is the assumption of a single scattering event. While a simple model that does not incorporate multiple scattering phenomena allows us to demonstrate the relevance of beam orientation optimization, multiple scattering might have a significant effect in large objects, so it would likely have some effect on optimization results. Future refinements of the model should incorporate a model of the multiple scattering contributions.

The process of modeling smearing due to detector inaccuracies using convolution of a triangular function roughly demonstrates that the scattering signal will not be a step function, but the amount of smearing was not directly calculated in relation to actual values seen in current detectors. While this does not affect optimization results where a single detector with consistent pixel size and a single energy resolution is used, model improvements could include calculations of additional smearing from detector components such as energy resolution and pixel size to provide a more accurate representation of the extent of expected smearing.

Additional validation of this model will involve computer simulation and experimental work. Experimental work regarding the effects of target location and object thickness on SAXS scattering signal has been previously performed by Dahal et al. [15]. This work involved the use of phantoms of various thickness with targets placed at 3 locations within the phantom. PMMA phantoms with thicknesses of 1, 3 and 5 cm were used. Amyloid targets were placed at the front, middle, and back of the PMMA phantoms, and SAXS data was acquired for each case. The results of this work provide a guideline for the experimental work that will provide further validation of the model. Larger objects could also be used to test the validity of certain assumptions of the model, including the neglecting of multiple scattering events.

More advanced phantom validation methods could involve an anthropomorphic head phantom with embedded amyloid targets of different sizes and at various locations to validate the effect of location on scattering profile. Challenges associated with these experiments include the need for external validation techniques, particularly when confirming the uniform density of amyloid within target regions.

We plan to utilize a variety of Monte Carlo simulation tools [16] to generate more accurate descriptions of the X-ray transport to further study and validate the beam orientation optimization methods described here. Simulations can be performed on 3D models of small-animal and human heads with amyloid targets at various locations within the objects. Comparison of simulation results to the results obtained with the described model will aid in the validation of the method as well as in providing a more accurate description of the radiation dose distribution in the different irradiated regions.

## Conclusion

Our findings support the relevance of beam orientation optimization for coherent X-ray scattering and provide a first-approximation estimate for selecting a strategy based on geometry and physics. Beam orientation optimization for coherent X-ray scattering will allow for determination of the optimal location and angular incidence to irradiate a target using an X-ray pencil beam.

## Methods

### Model assumptions

To interrogate deep targets in large objects, SAXS systems would benefit from using a medical-grade polyenergetic X-ray tube. However, for simplicity, this model assumes the use of a monoenergetic X-ray beam.

Multiple scattering occurs when photons are scattered more than once. The effect could remove quanta that suffered a small-angle coherent scattering event from the direction of interest or add spurious scattering signal coming from other areas within the object. This phenomenon occurs more frequently in larger objects since there are more opportunities for additional scattering events, and while typically ignored in small-sample SAXS measurements, could become more significant for larger objects. This model does not take into account multiple scattering phenomena that may occur and assumes a single scattering event in the path from source to detector. While a model that incorporates multiple scattering phenomena would be more accurate, our proposed model should effectively and rather simply demonstrate the importance of beam orientation optimization and provide a first approximation to the optimal solution.

Current X-ray detectors have limitations as they cannot locate and count photons with perfect accuracy at a rapid pace [17] resulting in a degradation of the signal and a spreading of the scattering angular data. A non-uniform density of amyloid scatterers can be modeled by setting the intensity of the scattering to be proportional to the density at each point within the target. For simplicity, this model is presented using a uniform density of scatterers throughout the target, since a uniform density sufficiently demonstrates the effectiveness of this model for beam orientation optimization. The density of scatterers outside the target is assumed to be zero. Linear attenuation coefficients vary within the human head, but for simplicity, the model assumes a uniform linear attenuation coefficient. Both the target and the object are assumed to have the same linear attenuation coefficient. If the differences in linear attenuation coefficient in the brain are drastic enough, optimization results could be affected, but in general, the assumption of one linear attenuation coefficient should effectively demonstrate beam orientation optimization. The probability of photon scatter is assumed to be the same at each point along the target diameter.

Solid angle effects were not included in the model as changes in solid angle are not significantly affected about a small scattering vector angular range. For each beam orientation optimization setup, we assumed a single type of detector would be used. Therefore, factors such as detector pixel size and energy resolution would be similar within each optimization problem and do not need to be included in the model under the assumption of a single detector with consistent pixel size and energy resolution.

### Geometrical considerations

The scattering vector *q* is defined as $q = 4 \pi sin\theta / \lambda$, where 2$\theta$ is the scattering angle and $\lambda$ is the wavelength. The relative uncertainty in *q* can be calculated using geometry [18], demonstrating the spread of *q* values based on target size, where $\theta$ is half the scattering angle, $L_0$ is the mean sample-to-detector distance, and $r_t$ is the target radius, as follows:

1 $$\begin{aligned} \frac{\Delta q}{q} = 2 - \frac{2}{sin \theta \sqrt{\left( \frac{L_0 + r_t}{L_0 tan \theta } \right) ^2 + 1}} \text {.} \end{aligned}$$

### X-ray physics

**Fig. 6:**
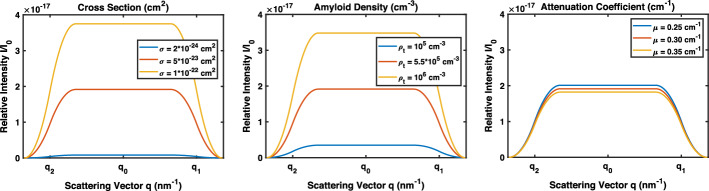
Relative scattering intensity is affected by the scattering cross-section, density of scatterers, and linear attenuation coefficient. Except when that variable is being manipulated, the value of the scattering cross-section for each plot is $5*10^{-23}$ $\text{cm}^{2}$, the amyloid density is $5.5*10^{5}$ $\text{cm}^{-3}$, and the linear attenuation coefficient is 0.30 $\text{cm}^{-1}$. The spherical target size $r_t$ is 0.5 cm and target-to-detector distance $L_0$ is 20 cm

The relative intensity of scattering is related to the the density of scatterers along the X-ray path, attenuation due to the size of the object, and the total scattering cross-section. This can be expressed as follows:

2 $$\begin{aligned} \frac{I}{I_0} = \int \limits_{-R_O}^{+R_O} \sigma \text{d}x \rho _t(x) e^{-\mu l} \text {,} \end{aligned}$$

where $\frac{I}{I_0}$ is the relative intensity, $\mu$ is the linear attenuation coefficient for gray/white matter, $\rho _t$ is the volumetric amyloid number density of the target, *l* is the total path length of the X-ray beam through the target, and $\sigma$ is the differential scattering cross-section. Figure 6 (middle) demonstrates how different values of scattering cross-section, amyloid density, and linear attenuation coefficient affect scattering signal.

The target and the object have the same linear attenuation coefficient, which is estimated using values for grey/white matter from ICRU report 44 [19] as referenced in the NIST X-ray attenuation databases.

There is a constant probability of scatter, as represented by the differential scattering cross-section, which was estimated using the scattering angle corresponding to amyloid, and coherent scattering molecular form factors in the range of 5–40 [20].

**Fig. 7:**
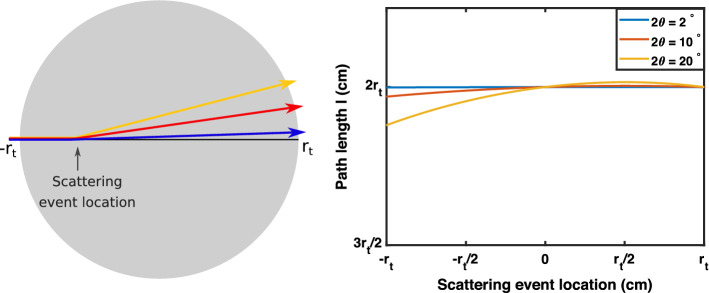
Path length of an X-ray pencil beam through a spherical samples changes based on the location of a scattering event. Path length is equal to the total distance traveled through the sample by the incoming ray and scattered ray

Path length was calculated by combining the length of incoming and scattered rays. In a spherical target, the length of the incoming ray was the distance traveled into the sample before scattering, and the scattered ray length was calculated using the law of sines. The relationship between scattering location and path length is shown in Fig. 7. Based on the results, we concluded that scatter event location does not have a significant effect on the total path length through the object, since the scattering angle $2 \theta$ is small. Thus, path length can be estimated using the path of the non-scattered X-ray beam.

When using SAXS to achieve a scattering signal from a target, the scattering signal from background objects is subtracted from the original scattering signal to isolate the scattering signal of scatterers in the target region. This ensures that the background-subtracted scattering signal is due to scatterers in the target region. This model represents a background-subtracted scattering signal, where all scattering originates from scatterers in the target region.

Additionally, scattering from brain matter generally does not occur in the same scattering vector range as amyloid scattering. There is minimal overlap with the scattering signal from the lipid content of brain matter [13].

The scattering profile described by Eq. 1 and Eq. 2 results in a step function with a height based on the relative intensity and width based on the uncertainty in q, and it represents the scattering profile obtained using an ideal photon counting detector. In order to more accurately model scattering behavior of an X-ray pencil beam captured by a photon counting detector, we must address the noise introduced into the system by degradations in the detector signal. The original scattering profile underwent convolution with a triangular function, $\Lambda$, with unit area and a width 0.35 times the spread of *q*.

**Fig. 8:**
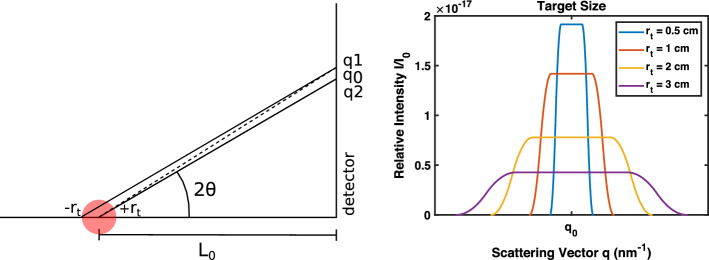
Target size affects scattering patterns for differing target sizes. Target size $r_t$ varies from 0.5 to 3 cm and mean target-to-detector distance $L_0$ is 20 cm

Figure 8 demonstrates the scattering signal modeled for spherical targets of different radii. The model determines the spread of the scattering vector using Eq. 1 and the relative scattering intensity based on Eq. 2. The blurring of the scattering profile representing the noise introduced by the detector has been introduced into the model using a convolution with a triangular function. This model demonstrates that as the target radius increases, peak relative intensity decreases, and uncertainty in *q* increases.

### Figure of merit

A figure of merit is developed to characterize the effectiveness of an X-ray path through a target for the purposes of our technique. Since scattering peaks are more easily distinguishable when they have higher scattering intensity, large numbers of photon counts, and less smearing of the scattering vector *q*, the figure of merit ($\Omega$) is described by

3 $$\begin{aligned} \Omega = \frac{I_{p} I_t}{l\Delta q} \text {,} \end{aligned}$$

where $I_p$ is the peak relative intensity of the scattering profile, $I_t$ is the total relative intensity determined by the area under the scattering profile curve, $\Delta q$ is spread of *q* given by the half-width-at-half-maximum, and *l* is path length through the object.

Radiation dose is an important factor to consider when determining the optimal X-ray beam path for a patient, since excessive radiation can cause damage to radiosensitive areas. Radiation dose is related to path length and tissue radiosensitivity. Since the cornea, optic lens, and oral cavity have high radiosensitivities, the optimal X-ray beam path should not intersect any of these areas, so beam orientations that intersect the cornea, optic lens, or oral cavity are not considered. Bone and neuronal tissue have similar and fairly low radiosensitivities, so further differences in radiosensitivity are not considered, and radiation dose is assumed to be proportional to *l*. Radiation dose was incorporated into the figure of merit by including path length *l*.

## Data Availability

Not applicable.
